# Supplementary material for: Mechanisms governing the pioneering and redistribution capabilities of the non-classical pioneer PU.1
Source: Nat Commun. 2020 Jan 21;11:402. doi: 10.1038/s41467-019-13960-2 (PMC6972792; doi:10.1038/s41467-019-13960-2)
Supplement: Supplementary file 7 — Source data [file 41467_2019_13960_MOESM7_ESM.zip › Source_Data/FigureS3/FigureS3J_MotifScanOutput/homerResults/motif67.info.html]

Motif 67

## Information for 25-TATTTACCA (Motif 67)

A
C
G
T
C
G
T
A
A
C
G
T
A
C
G
T
A
C
G
T
C
G
T
A
A
G
T
C
A
G
T
C
C
G
T
A
  
Reverse Opposite:
